# Wnt/ß-Catenin: A New Therapeutic Approach to Acute Myeloid Leukemia

**DOI:** 10.4061/2011/428960

**Published:** 2011-12-01

**Authors:** Y. Kim, S. Thanendrarajan, I. G. H. Schmidt-Wolf

**Affiliations:** Department of Internal Medicine III, Center for Integrated Oncology (CIO), University of Bonn, Sigmund-Freud-Straße 25, 53105 Bonn, Germany

## Abstract

Recent studies have shown genetic and epigenetic aberrations resulting in aberrant activation of the Wingless-Int (Wnt) pathway, thus influencing the initiation and progression of acute myeloid leukemia (AML). Of major importance, these findings may lead to novel treatment strategies exploiting targeted modulation of Wnt signaling. This paper comprises the latest status of knowledge concerning the role of Wnt pathway alteration in AML and outlines future lines of research and their clinical perspectives.

## 1. Acute Leukemia

Acute myeloid leukemia (AML), a hematologic malignancy of the myeloid line of white blood cells in the bone marrow, is the most common leukemia of the adult [1, 2]. Despite major progress in the treatment of AML during the last couple of years, the majority of patients still cannot be cured. Different genetic causes result in variable clinical courses of AMLs and different responses to standard chemotherapy including stem cell transplant [1, 3, 4].

Due to high mortality rates and high relapse rates even among transplanted patients, new therapeutic strategies are required [4, 5].

The Wingless-Int (Wnt)/beta-catenin pathway has been shown to play an essential role in the regulation of cell proliferation, differentiation, and apoptosis of haematopoietic stem cells [1, 2, 4, 5]. Recently, it was demonstrated that deregulation of this pathway resulted in different malignancies including AML. Thus, Wnt/beta-catenin signaling molecules are attractive candidates for developing novel targeted therapies for this disease [1–4, 6].

## 2. Wnt Signaling Pathway

Wnt (wingless) proteins constitute a family of cysteine-rich glycosylated proteins that contribute to lymphopoiesis and early stages of both B-cell and T-cell development [7–10]. They function as extracellular signaling molecules that may activate the Wnt/beta-catenin signaling pathway by binding to the extracellular domain of Frizzled receptors. In addition to their extracellular Wnt-binding domain, Frizzled receptors have seven transmembrane-spanning sequences and a C-terminal tail [10–12]. Wnt proteins regulate cell proliferation, cell morphology, cell motility, and cell fate. 

To date, 19 Wnt members have been identified in humans and more than eight mammalian Frizzled genes are known [11–13]. Wnt signaling results in the activation of intracellular signaling cascades which are associated with several forms of cancer [11, 14].

Binding of Wnt either to Frizzled and the low-density lipoprotein receptor-related proteins (LRPs) 5 and 6 or to Frizzled protein alone results in the stabilization of beta-catenin, the major mediator of canonical Wnt signaling [13, 14]. Frizzled receptors have no enzymatic motifs on their intracellular domains, therefore intracellular signaling molecules have to be recruited or released [12]. These are members of the Dishevelled (Dvl) family. 

Three members of Dvl, Dvl-1, Dvl-2, and Dvl-3, have been characterized [15]. They lack any known enzymatic activity but function as molecular adaptors on Frizzled receptors due to their protein-protein interaction domains and/or heterotrimeric G-proteins [12, 14]. Beta-catenin is associated in a cytoplasmic complex together with the adenomatous polyposis coli (APC) protein, the cytoplasmic serine/threonine kinase GSK-3beta, and axin [12, 16]. 

In unstimulated cells, axin in its phosphorylated form is able to bind beta-catenin effectively. Upon Wnt signaling, however, axin is dephosphorylated by the protein phosphatase 2A (PP2A). The dephosphorylated form of axin binds beta-catenin less efficiently than it does the phosphorylated form, thereby promoting the release of beta-catenin [17]. A complex of axin with one of the three isoenzymes of casein kinase I (CKI*α*, *δ*, or *ε*) phosphorylates beta-catenin on serine 45. This step is independent of (GSK-3beta) and initiates the phosphorylation-degradation cascade of beta-catenin. In the following step, beta-catenin is phosphorylated at serine 33/37 by GSK-3beta [15].

 The phosphorylated beta-catenin is, subsequently, recognized by the E3 ubiquitin ligase subunit beta-transducin repeat-containing protein (beta-TrCP) and thereby targeted for ubiquitination and subsequent degradation by the proteasome [16, 18, 19].

In the presence of Wnt signaling, the phosphorylating activity of GSK-3beta is inhibited leading to the stabilization of beta-catenin [20]. This is mediated by the intracellular protein Dvl through a Frizzled receptor [16]. The APC-GSK-3beta-axin activity is dissociated and unphosphorylated beta-catenin accumulates in the cytoplasm [16, 20]. From there, free beta-catenin is able to translocate into the nucleus where it interacts with TCF and LEF transcription factors [20, 21]. 

Dvl not only stabilizes beta-catenin in the cytoplasm but is also required in the nucleus where it interacts between c-Jun and beta-catenin, respectively. This mediates the formation of a Dvl-c-Jun-beta-catenin-TCF transcriptional complex, binding on the promoter of Wnt target genes [14]. For example, beta-catenin may stimulate cell-cycle progression and differentiation by Wnt/beta-catenin target gene expression of genes, including *c-myc*, *cyclin D1,* and* fibronectin* [11, 14, 19]. 

## 3. Beta-Catenin

Beta-catenin is a 92 kDa protein consisting of several structural domains [12, 21–23]. The N-terminal region mediates binding activity by phosphorylation sites for glycogen synthase kinase-3beta GSK-3beta and beta-catenin [12, 20, 24]. The central domain contains 13 incomplete conserved Armadillo repeat motifs facilitating protein-protein interactions of, for example, cadherins, beta-catenin, the APC protein, axin, or lymphoid-enhancing-transcription-factor-(LEF-) 1/T-cell transcription factor (TCF) [12, 21, 24, 25]. A positively charged groove in a superhelix of this central domain is hypothesized to interact with acidic regions of APC, TCF transcription factors, and cadherin cell-adhesion molecules [12, 26–28]. The C-terminal region encodes a transcriptional transactivation domain. Both the central domain and the C-terminal region are involved in the signaling activity of beta-catenin [21, 29–31]. 

Beta-catenin acts as a structural protein at cell-cell adherens junctions where it links cadherins to the actin cytoskeleton [32–34]. In addition to beta-catenin, there are two other catenins known as alpha-catenin and gamma-catenin [35–37]. Beta-catenin associates with E-cadherin and alpha-catenin, forming a cadherin-catenin protein complex under **in vivo **conditions [21, 38]. Furthermore, beta-catenin acts as a central molecule in the Wnt pathway, influencing membrane structure and the shape of a cell [11, 16, 39]. Thus, beta-catenin has a dual cellular function in mediating cell-cell adhesion as well as Wnt signaling [21, 40, 41].

## 4. Wnt Pathway Signaling in AML

The Wnt/beta-catenin pathway has been shown to play a critical role in the regulation of cell proliferation, differentiation, and apoptosis [1, 4–6] of different malignant entities. It is highly regulated in AML and also involved in the self-renewal process of hematopoietic stem cells [5, 7, 8].

It was recently published that AMLs are associated with an aberrant activation of the Wnt pathway, thus representing an attractive way of targeted therapeutic intervention [3]. However, the exact mechanism in the physiology of leukemia cell lines and the impact of the Wnt pathway on the course of the disease *in vivo* still need to be clarified [4, 6].

Within the canonical Wnt signaling pathway, beta-catenin acts as the major effector molecule affecting self-renewal of hematopoietic stem cells while its dysregulation has been suggested in leukemic stem cell lines [3, 6–8]. Aberrant activation of this pathway can lead to a variety of malignant abnormalities with one of them being acute myeloid leukaemia. Recent published data proved that reduction of intracellular beta-catenin levels in AML cell lines and patient samples decreased their rate of proliferation *in vitro* without affecting cell viability in contrast to what is known for normal human CD34+ progenitor cells [2, 4, 5, 10]. It was shown that downregulation of beta-catenin caused a G1/G2 phase increase of cells while lowering the amount of S phase cells, which has recently been reported for multiple myeloma [6]. Other data, however, suggests that, despite effective downregulation of beta-catenin in primary AML cell lines, a decreased proliferation rate could not always be observed [7, 8, 10]. Thus, targeted therapy against beta-catenin might not be successful in all patients [5, 7].

Overexpression of beta-catenin seems to be an independent adverse prognostic factor, its overregulation could be found in the vast majority of AML samples [2, 4, 8]. In a recently published study the intracellular concentration of beta-catenin was downregulated by a short hairpin RNA lentivirus deteriorating the proliferation of leukemic cell lines, even resulting in a reduced engraftment potential after xenotransplantation [1, 4, 5]. Interestingly, in several studies, beta-catenin expression did not always correlate with AML subtype or karyotype, which was possibly due to a low number of investigated patients and cell lines [5, 6]. These published data support the fact that, in most AML samples, Wnt/b-catenin is overregulated and that this is a feature shared by leukemic stem cells regardless of their CD34 status [8, 9].

We recently identified several drugs as Wnt inhibitors [11, 12, 42, 43]. It was shown that downregulation and inhibition of the Wnt/beta-catenin pathway significantly reduced tumor cell viability in lymphoma and myeloma cell lines *in vitro*. Systemic application of these drugs in balb/c mice resulted in a decreased tumor growth and a prolonged overall survival [42, 43]. These data could be used to establish further drug studies, in AML cell lines.

All these data greatly contribute to our understanding of the role of beta-catenin and the Wnt pathway in the development of AML. The canonical Wnt pathway could be shown to be of major importance in the pathogenesis of acute leukaemia. In spite of divergent results in recent studies influencing this pathway seems to be a promising treatment strategy and should be followed up in further studies for future clinical use in patients with AML.
